# Development and application of reverse genetic technology for the influenza virus

**DOI:** 10.1007/s11262-020-01822-9

**Published:** 2021-02-02

**Authors:** Ziquan Li, Liping Zhong, Jian He, Yong Huang, Yongxiang Zhao

**Affiliations:** National Center for International Research of Bio-targeting Theranostics, Guangxi Key Laboratory of Bio-targeting Theranostics, Collaborative Innovation Center for Targeting Tumor Diagnosis and Therapy, Guangxi Medical University, Nanning, 530021 Guangxi China

**Keywords:** Influenza virus, Reverse genetic technology, Vaccine, Antitumor

## Abstract

Influenza virus is a common virus in people's daily lives, and it has certain infectivity in humans and animals. Influenza viruses have the characteristics of a high mutation rate and wide distribution. Reverse genetic technology is primarily used to modify viruses at the DNA level through targeted modification of the virus cDNA. Genetically modified influenza viruses have a unique advantage when researching the transmission and pathogenicity of influenza. With the continuous development of oncolytic viruses in recent years, studies have found that influenza viruses also have certain oncolytic activity. Influenza viruses can specifically recognize tumor cells; activate cytotoxic T cells, NK cells, dendritic cells, etc.; and stimulate the body to produce an immune response, thereby killing tumor cells. This article will review the development and application of influenza virus reverse genetic technology.

## Introduction

Genetics is traditionally defined as the phenotype and traits of an organism and represents a branch of science that studies the genetic composition of organisms, whereas reverse genetics is a process designed to identify the genetic composition of an organism and investigate the phenotype and traits at genetic level [1]. Reverse genetic technology is applied through targeted transformation of biological genes, such as by site-directed mutation, base insertion, deletion, replacement, etc., and then the transformed gene is modified and assembled so that it can be stably expressed [2, 3].

Influenza virus belongs to the genus Orthomyxovirus of the Orthomyxoviridae family which includes enveloped segmented single-stranded negative-sense RNA viruses [4]. Reverse genetic technology uses modified cloned cDNA to obtain infectious viruses to study the effect of these modifications on phenotypes. The reverse genetic system of influenza A virus was established by Luytjes and Enami in 1989 and 1990, respectively [5, 6]. In 1993, Takizawa discovered that influenza virus had a certain proapoptotic effect on MDCK and HeLa cells cultured in vitro and proposed for the first time that influenza virus could induce apoptosis in cultured cells in vitro [7]. In 1997, a positive signal of apoptosis was observed after a mouse was infected with influenza virus in the nasal cavity, and this signal showed that influenza virus can also cause apoptosis in the body [8]. Since then, the continuous development of reverse genetic technology has had a massive impact on the study of the transmission characteristics, infection mechanisms, and antitumor mechanisms of influenza viruses and the development of influenza vaccines. This article will review and discuss the reverse genetic technology of influenza virus and its research.

## Introduction to influenza virus

**Fig. 1:**
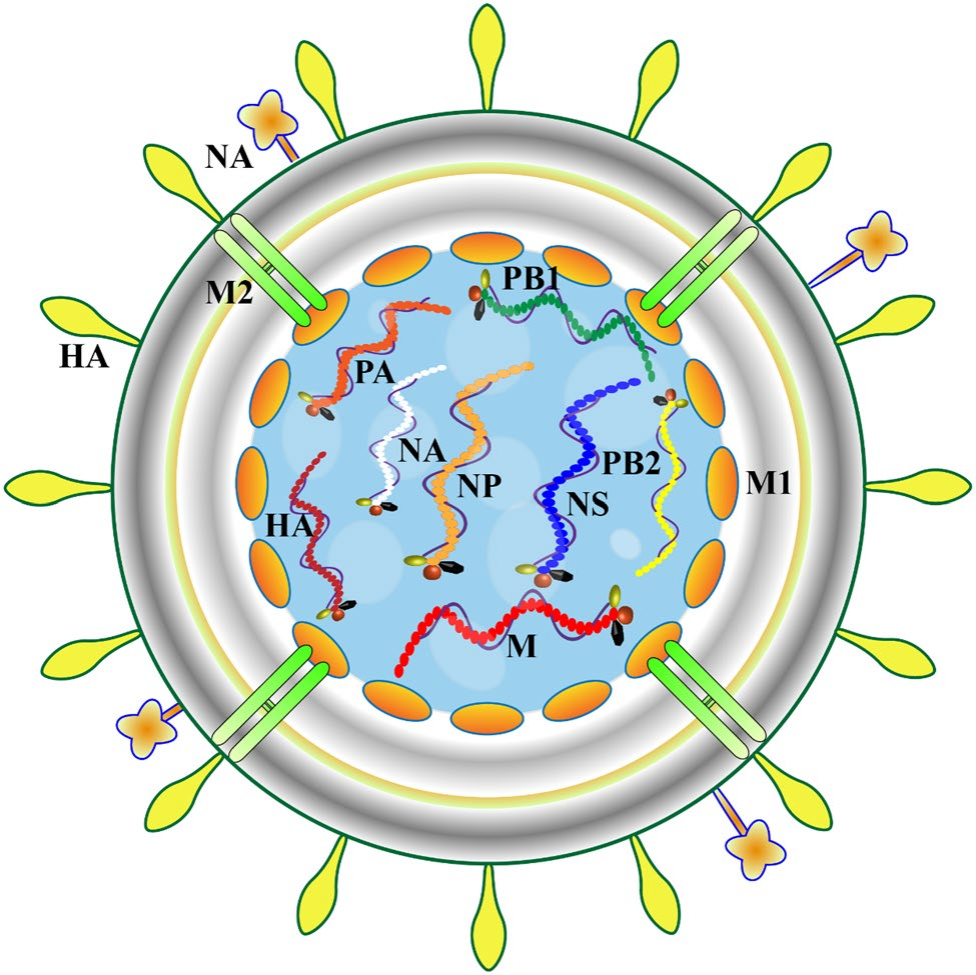
Schematic diagram of the influenza virus structure. *HA* hemagglutinin, *NA* neuraminidase, *M1* matrix protein 1, *M2* Matrix protein 2, *PB1* polymerase basic protein 1, *PB2* polymerase basic protein 2, *NS* nonstructural protein, *NP* Nucleoprotein, *PA* polymerase acidic protein

Influenza viruses are spherical, and newly isolated viruses are generally 80–120 nm in size and consist of mostly filamentous particles up to 4000 nm [9]. The viral genome is approximately 13.6 kb and divided into 8 independent fragments of varying sizes. The 8 genome fragments encode the 8 structural proteins (PB1, PB2, PA, HA, NP, NA, M1, and M2) and nonstructural proteins (NS1 and NS2) of influenza virus [10] (Fig. 1). Because the nucleoprotein (NP) of influenza virus is highly conserved, it can be divided into three types: A, B, and C [11]. Influenza A virus is the main research object of this article. In addition to the above 10 essential proteins, the influenza A genome also encodes up to 7 nonessential accessory proteins (PB1-F2, N40, PA-X, PA-N155, PA-N182, M42, and NS3) [12–15]. Influenza A virus contains hemagglutinin (HA) and neuraminidase (NA). According to their antigenicity, the virus can be divided into 18 H subtypes and 11 N subtypes.

## Main functional proteins of influenza virus

The influenza virus genome encodes a variety of proteins, including proteins that play a decisive role in the replication, spread, infection, and pathogenicity of the virus. The polymerase of influenza A and B viruses is a complex of three proteins, namely, polymerase basic protein 1 (PB1), polymerase basic protein 2 (PB2) and polymerase acidic protein (PA). These proteins bind to one end of an antiparallel duplex formed by viral RNA and nucleoprotein (NP) and together form a viral ribonucleoprotein (vRNP) complex [16–19]. After virus infection, the vRNP is transported to the host cell nucleus and the modified viral RNA produces cRNA and mRNA. The former is a template for progeny viral RNA replication, and the latter is a template for viral protein translation [20].

After infection by the virus, HA binds to the sialic acid receptor on the surface of the host cell and the virus invades the cell through receptor-mediated endocytosis. In addition, HA will also promote the fusion of the viral capsule and the host cell membrane [21–23]. HA will produce the polypeptide chain HA0 after translation, which is cleaved by host proteases into the HA1 and HA2 subunits, which become active HA proteins. HA1 is responsible for binding to the receptor, and HA2 is responsible for membrane fusion [24–26]. The main function of the NA protein of influenza virus is to cleave sialic acid on the surface of host cells or on the surface of newly generated virus particles. The NA protein can prevent the accumulation of virus particles on the cell surface and promote the release of new virus particles from the host cell after budding [27]. However, studies have shown that the sialidase activity of NA protein can also help viruses enter host cells [28]. In addition, the ratio of HA to NA on the virus particles can hinder viral passage through the receptor-rich mucus layer and ultimately affects the ability of the virus to infect host cells [29, 30].

The matrix protein 2 (M2) of influenza virus is a multifunctional modular protein. M2 constitutes the proton channel of the virus. The proton channel undergoes a conformational change in the acidic environment of intracellular vesicles, thus causing the protons in the intracellular vesicles to enter the virus particles through the proton channel. This activity promotes the fusion of the viral capsule with the intracellular vesicle membrane and the release of the vRNP complex into the cytoplasm [31, 32]. M2 can also stabilize the pH in the cytoplasm, prevent the conformational changes of HA, and promote the release of progeny viruses [33].

The matrix protein 1 (M1) of influenza virus is crucial for the assembly of virus particles, and M1 is also the main determinant of the shape of the virion [34]. Nonstructural protein 2 [NS2, also known as nuclear export protein (NEP)] mainly mediates the transport of vRNP from the nucleus to the cytoplasm. Recent studies have shown that the expression of NS2 is essential for determining the level of virus replication [35]. Nonstructural protein 1 (NS1) is an antagonist of the host's natural immune response induced by the virus [36]. The NS1 protein can inhibit the production of interferons in the host and the establishment of antiviral status. Similarly, NS1 also controls the synthesis and splicing of viral RNA, as well as restricts the host cell's mRNA polyadenylation [35].

## Life cycle of influenza virus

**Fig. 2:**
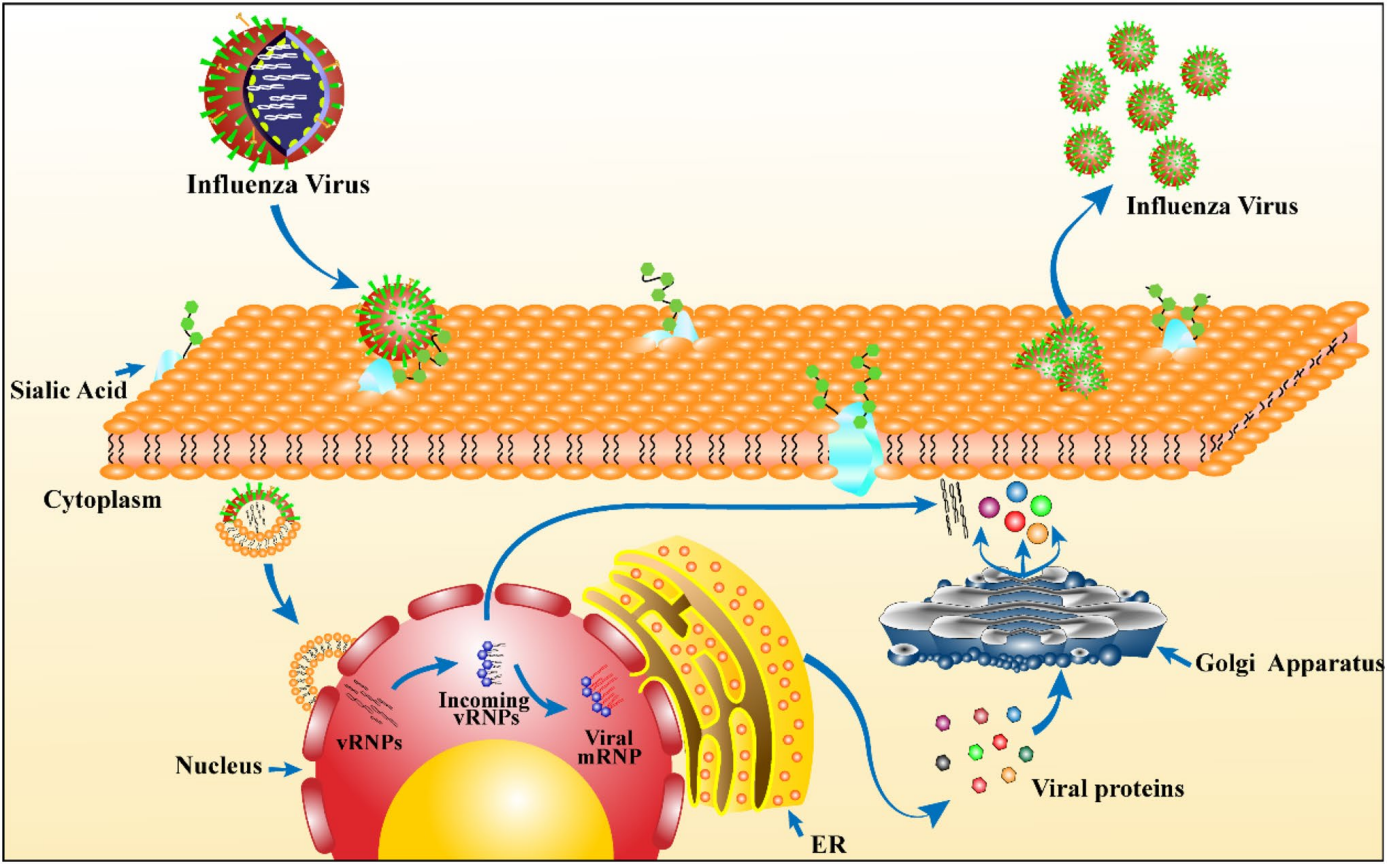
Influenza virus infection of host cells. The HA of the influenza virus binds to the sialic acid-terminating glycan receptor on the surface of the host cell and enters the host cell through endocytosis. The vRNP released by the virus enters the host cell nucleus and produces viral mRNA. By translating the newly generated viral protein and the progeny vRNP, the progeny virus is synthesized again and released

Influenza virus replication refers to the whole process of virus particle invasion into the host cell to the final cellular release of the progeny virus, including adsorption, entry, uncoating, gene expression, assembly, and release (Fig. 2). Influenza viruses bind to sialic acid-terminating glycan receptors on the surface of host cells through HA. The vRNP complex is released into the cytoplasm through endocytosis. Low-pH conditions stimulate HA2-mediated membrane fusion and activate the M2 proton channel, thus accelerating the release of vRNP into the host cytoplasm [37]. The newly synthesized vRNP interacts with the M1 protein, is released outside the nucleus with the participation of the NS2 protein, and is incorporated into the progeny virus particles containing HA, NA and other proteins. The progeny viruses are then released outside the host cell through the cell membrane [38, 39].

## Overview of reverse genetics of viruses

**Fig. 3:**
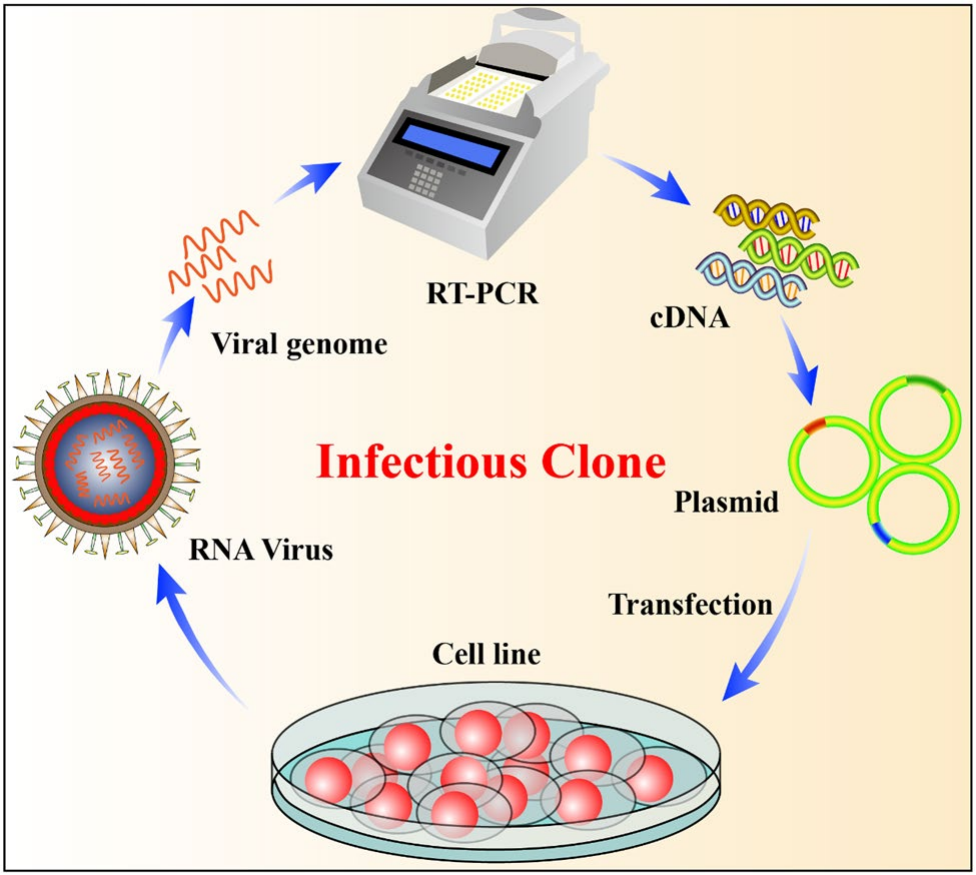
Process of viral rescue. Viral RNA is extracted and reverse- transcribed into RNA viral genomic cDNA by RT-PCR. The cDNA is inserted into the vector plasmid, and the recombinant virus is obtained by transfecting cells in vitro

The reverse genetics of viruses is often called viral rescue, which is achieved by constructing an infectious clone of the virus and manipulating it in vitro at the DNA level to study the structure and function of the virus [40, 41]. Infectious molecular clones include infectious cDNA and infectious in vitro transcripts. To construct infectious cDNA, the cDNA fragment of the RNA virus genome is amplified by reverse transcription-polymerase chain reaction (RT-PCR), and restriction enzyme sites are used to clone it into a suitable vector to obtain a full-length cDNA clone of the genome. Using this clone to transfect appropriate cells, the full-length cDNA is replicated and transcribed, and it produces all the components of the virus in the cell and finally packages them into infectious virus particles [42, 43] (Fig. 3).

## Development of influenza virus reverse genetic technology

Viruses can be divided into DNA viruses and RNA viruses according to their genome types. RNA viruses can be divided into positive-sense RNA viruses and negative-sense RNA viruses. When the whole genome RNA of a positive-sense RNA virus is transfected into eukaryotic cells, its RNA can directly serve as mRNA [44, 45]. The genome of a negative-sense RNA virus cannot be used directly as a template for viral protein translation. To replicate normally, RNP complexes must be formed [46].

In 1976, the first reverse genetic manipulation system for DNA viruses was established. Goff et al. successfully rescued in vitro DNA containing the artificial mutation SV40 [47]. In 1978, Taniguchi et al. established the first non-segmented reverse genetic manipulation system for positive-sense RNA viruses. The first in vitro rescue of an RNA virus, the Qβ phage, was achieved, and the difficulty of RNA virus rescue was overcome [48]. It was not until 1989 that the Palese research team completed the manipulation of the genome of a negative-sense RNA virus for the first time, which became the starting point of reverse genetic research of negative-sense RNA viruses [5]. In 1994, Neumann et al. successfully achieved the use of the human RNA polymerase I system to transcribe RNA fragments of influenza virus in cells, which laid the foundation for the establishment of the reverse genetic system of influenza virus [49]. Subsequently, the scientists used the T7 RNA polymerase system to establish a reverse genetic system for a plasmid-based, non-segmented negative-sense rabies virus [50]. In the next few years, scientists used similar methods to establish a variety of reverse genetic systems of non-segmented negative-sense RNA viruses, such as human respiratory syncytial virus, parainfluenza virus, Sendai virus, and rinderpest virus [51–54]. Building on the success of these practices, Bridgen and Elliott used the T7 RNA polymerase system to establish a reverse genetic system for Bunya virus. The Bunya virus genome is a negative-sense RNA virus divided into 3 segments [55].

**Fig. 4:**
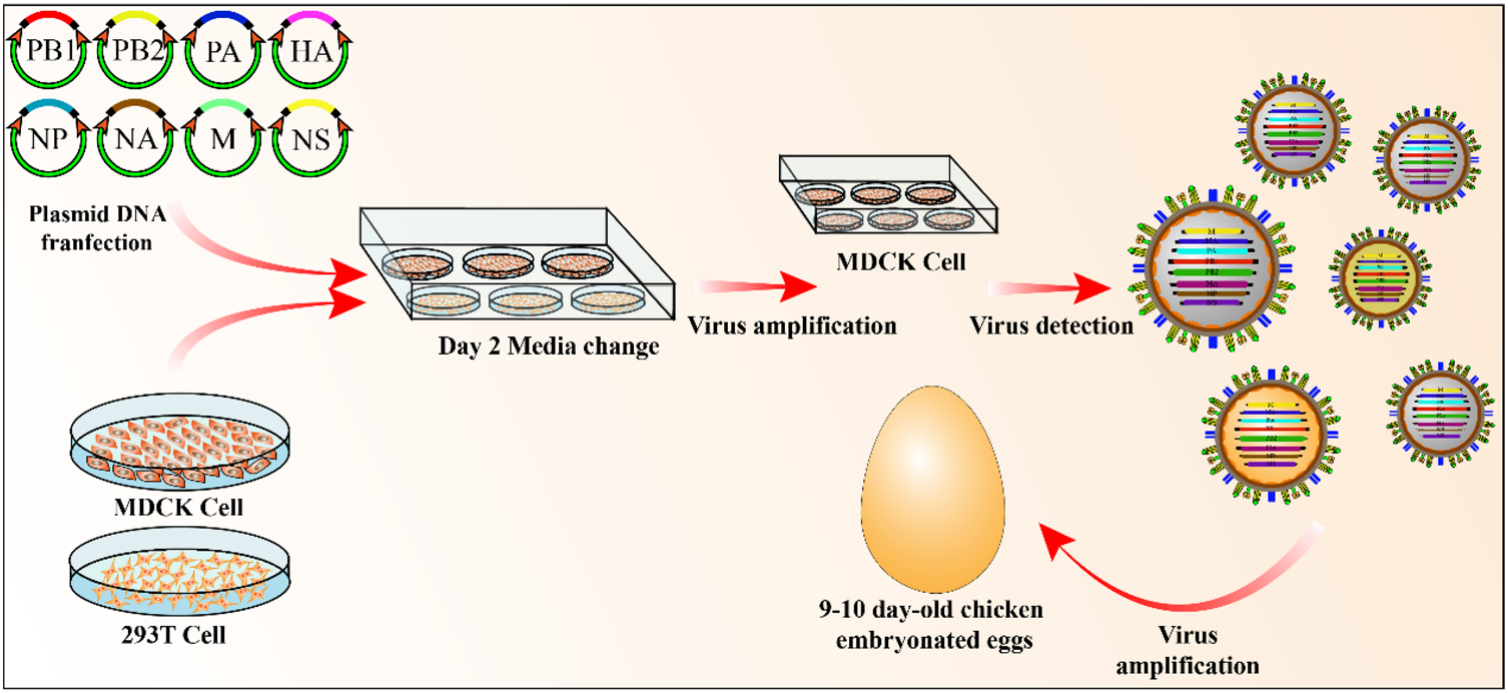
Schematic diagram of the influenza virus 8-plasmid reverse genetic operation process. MDCK and 293 T cells are co-transfected with plasmids containing 8 influenza virus gene fragments. MDCK cells are infected to identify whether the recombinant influenza virus can be successfully rescued. A large amount of recombinant virus is amplified by infected chicken embryos

In 1999, Neumann et al. of the Kawaoka research team established for the first time a completely plasmid-based reverse genetic system for influenza viruses. This system clones the cDNAs of eight kinds of RNA fragments of influenza virus into a plasmid vector containing the human RNA polymerase I promoter and mouse RNA polymerase I terminator one by one. The genes encoding nine viral proteins (PB2, PB1, PA, HA, NP, NA, M1, M2, and NS2) were cloned into the protein expression vector containing the RNA polymerase II promoter and poly-A signal one by one. For the first time, this study eliminated the large amount of screening processes required for auxiliary viruses, which was a large step toward the rescue of influenza viruses in vitro [56]. Since then, Fordor et al. also established a reverse genetic system for influenza virus in 1999. Unlike the 17-plasmid system of Neumann et al. they used a 12-plasmid system to rescue influenza virus [57]. The so-called 12-plasmid system includes PB2, PB1, PA and NP, four RNA polymerase II expression plasmids, and eight RNA polymerase I expression plasmids. The cells are transfected with these plasmids, which transcribe and express the viral genomic RNA and virus-related proteins in the cells, thereby producing infectious virus particles. Based on the 12-plasmid system, Hoffmann et al. further established an 8-plasmid system of influenza virus reverse genetic manipulation technology in 2000 [58] (Fig. 4). The system further improved the RNA polymerase I system and led to the invention of the "bidirectional vector", which indicates that the cDNA encoding the influenza virus gene fragment is positively inserted between the RNA polymerase II promoter and the poly-A signal, and then the RNA polymerase I promoter and terminator sequences are inserted in reverse at both ends. This arrangement enables viral RNA replication and protein expression on the same template. Among them, RNA polymerase I is responsible for transcribing negative-strand viral RNA and RNA polymerase II is responsible for synthesizing positive-strand mRNA, thus reducing the 12 required plasmids to 8. Current technology is mainly based on the 8-plasmid system; however, obtaining viral RNA and expressing viral proteins on the same template reduces the "elasticity" of the system. As a result, when studying gene delivery or viral proteins, if one or more fragments are missing or if a certain fragment(s) has a lethal mutation, then the virus cannot be rescued.

## Latest progress of influenza virus reverse genetic technology

**Fig. 5:**
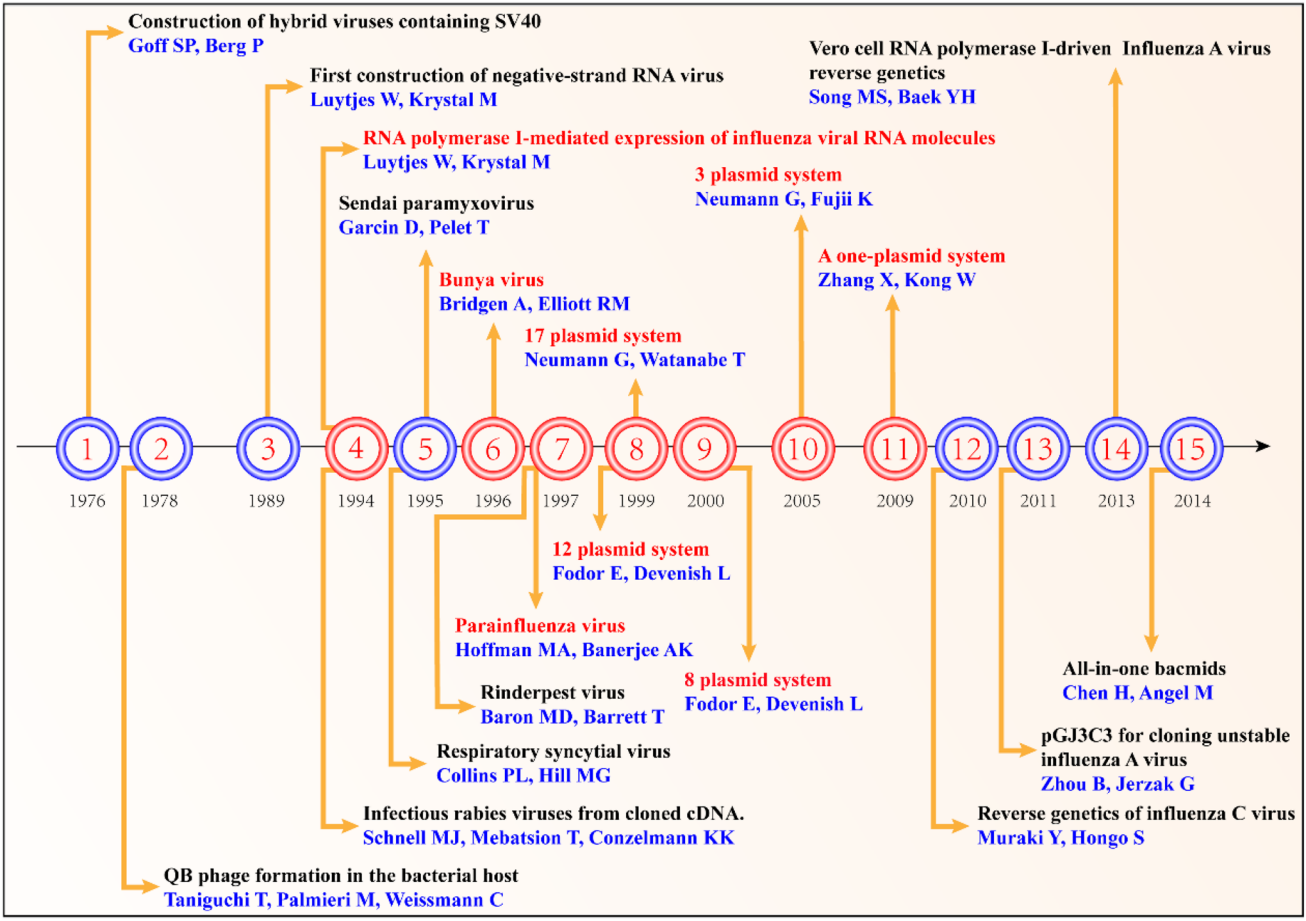
Influenza virus reverse genetic technology development timeline. The timeline is from 1976 to 2014

Based on the 12-plasmid system and the 8-plasmid system, Neumann et al. cloned the RNA polymerase I transcription units of all 8 gene fragments into one plasmid. The RNA polymerase II transcription unit expressing the three polymerase proteins PB2, PB1, and PA of the virus was cloned into another plasmid. In addition, the RNA polymerase II transcription unit expressing the viral nuclear protein NP was cloned into a plasmid. With this system, only three plasmids need to be transfected to rescue influenza virus. This system can greatly improve the efficiency of influenza virus rescue in cell lines with low transfection efficiency (such as Vero cells) [59]. Subsequently, Zhang et al. cloned the bidirectional transcription unit of all 8 gene fragments of influenza virus into a plasmid. Transfecting this plasmid in chicken embryo fibroblasts can efficiently rescue influenza viruses [60]. In 2010, Muraki et al. established an influenza C virus-like particle (VLP) generation system and reverse genetic system [61]. Zhou similarly constructed the low-copy plasmid pGJ3C3 in 2011. This plasmid can be used to clone other unstable segments of influenza A virus and rescue recombinant virus. This technology has promoted basic research and vaccine production of influenza A virus [62]. Song et al. constructed a reverse genetic system using the Vero cell RNA polymerase I promoter to replace the traditional human RNA polymerase I promoter. The results showed that the Vero polymerase I promoter transcription level in Vero cells and the rescue efficiency of human RNA viruses were improved compared with those for the reverse genetic system transfection of 293 T cells containing the human polymerase I promoter [63]. Subsequently, Chen et al. cloned the bidirectional transcription unit of 8 gene fragments of influenza virus into bcmd-RGFlu. After transfection, these fragments can efficiently rescue influenza virus in a variety of cells [64]. Although these new attempts have greatly reduced the number of plasmids required to rescue influenza virus, the 8 gene fragments of the virus need to be integrated into one or several vectors at the same time, which significantly increases the difficulty and complexity of the cloning process (Fig. 5).

## Reverse genetic technology use in influenza virus pathogenicity research

The application of reverse genetic technology has played an important role in the discovery of the factors that determine influenza virus pathogenicity. Studies have found that the low-pathogenicity avian influenza virus has only one alkaline arginine at the HA cleavage site and can be cleaved by trypsin-like proteases present in the respiratory and digestive tracts; therefore, the virus is generally restricted to replication in the respiratory and digestive tracts [65, 66]. The highly pathogenic H5 and H7 subtype avian influenza viruses have multiple consecutive alkaline amino acids at the HA cleavage site that can be cleaved by proteases widely present in cells, which potentially leads to systemic infection [67]. A reverse genetic system was used to remove multiple basic amino acids from the HA cleavage site of highly pathogenic avian influenza virus, and the results showed that the virus's pathogenicity in poultry and mice was reduced [68]. During the epidemic of H5N1 avian influenza virus, 20 amino acids at positions 49 to 68 were deleted from the stem of the NA protein. Viruses containing this deletion expanded significantly in 2002. By 2007, all H5N1 avian influenza viruses had acquired this deletion [69]. After rescue of the mutant virus containing this deletion by reverse genetic technology, it was found that the NA stem deletion virus was significantly more pathogenic than the wild-type virus. At the same time, using reverse genetic technology, multiple amino acid mutations affecting the pathogenicity of influenza virus were found in the NS1 protein [70–72].

## Influenza virus reverse genetics research for the development of new vaccines

The best method of preventing flu is vaccination. Therefore, new and more effective influenza vaccines are urgently required. The three common forms in global influenza vaccine development are inactivated influenza vaccines, recombinant influenza vaccines and live attenuated influenza vaccines [73]. The recombinant influenza virus constructed by plasmid-based reverse genetic technology is an important object in the research of recombinant influenza vaccines. Single or multiple viral genome mutations contained in this form can provide additional research methods for new or improved vaccines.

With the development of influenza virus reverse genetic technology, inserting foreign epitopes into influenza virus protein structural domains to exert a better immune function has become an extensive research method. The most representative influenza virus vaccine vector is the cold-adapted live attenuated influenza vaccine (LAIV), which has been approved for use in many countries [74–76]. The phenotype of the LAIV is controlled by multiple mutations in the internal protein genes of the donor virus. Therefore, the LAIV viral vector is relatively stable and the probability of reassortment with wild-type influenza virus is low [77, 78]. In addition, recombinant vaccine vectors modified for influenza virus proteins are also widely used. Vaccine vectors targeting HA and NS1 are the most common. The stalk domain of the HA protein is relatively conserved among different subtypes of influenza viruses; therefore, the development of an influenza vaccine against the stalk domain of the HA protein is a new approach to influenza vaccine development. Studies have confirmed that virus-like particles composed of the HA protein with the missing head and the HA protein stem domain antigen are widely effective in clinical trials [79, 80]. In addition, since NS1 can antagonize the host's innate immune type I interferon (IFN-I) response, a variety of potential vaccine strategies have been developed, and they are mainly based on the use of modified NS1 protein as a means of virus attenuation [81].

## Antitumor research using influenza virus reverse genetic technology

### Antitumor application of influenza virus

Although influenza virus is a pathogen that endangers human health, many studies have found that it has certain oncolytic ability as well. In 1993, Takizawa discovered that MDCK and HeLa cells cultured in vitro showed a series of specific changes in apoptosis after being infected with human influenza A virus, and he proposed for the first time that influenza virus can induce apoptosis in cultured cells in vitro [7]. In 1997, a positive signal of apoptosis was observed in the mouse nasal cavity after infection with influenza virus, indicating that influenza virus can also cause apoptosis in the body [8]. Afterward, an influenza virus vector with MAGE-3 constructed by Storbel et al. could transfect human dendritic cells (DCs) and highly express MAGE-3. The transfected DC phenotype does not affect its antigen presentation function and can stimulate the generation of MAGE-3-specific cytotoxic T lymphocytes (CTLs) [82]. These cells can induce an effective antitumor immune response. Sanda Sturlan et al. constructed replication-deficient influenza A virus, induced peripheral mononuclear cells to produce IFN-γ, activated CTLs, and induced CD8+ immune pathways to kill tumor cells [83]. R Weiss constructed an influenza virus vector expressing IL-24, which can enhance influenza virus-mediated apoptosis. Studies have found that the recombinant virus has stronger oncolytic activity than the virus alone and IL-24 [84]. Recently, Jennifer R. Hamilton et al. inserted anti-CTLA4 antibodies into the PB1 and PA segments of influenza virus and found that it had good therapeutic effects in mice with aggressive B16-F10 melanoma [85].

### Apoptosis and influenza virus

Typical features of apoptosis include DNA fragmentation, phosphatidylserine exposure on the cell membrane, plasma membrane deformation and blistering, and apoptotic body formation. Apoptosis can generally be divided into intracellular pathways and extracellular pathways. Influenza virus infection is a factor that causes apoptosis. It can cause apoptosis through intracellular pathways or extracellular pathways [86]. Studies have found that the PB1-F2 protein expressed by the influenza virus can interact with mitochondria in infected cells, change the permeability of the mitochondrial membrane, and accelerate the release of cytochrome c and the production of apoptotic bodies, thereby internally causing cellular apoptosis [87–89]. In addition, after influenza virus infects host cells, this protein also promotes the expression of Fas cell surface death receptor ligand (Fasl) and tumor necrosis factor-related apoptosis-inducing ligand (TRAIL) and other death receptor ligands, which externally cause cell apoptosis [90, 91].

### Immunity and influenza virus

The elimination of the virus by the human immune system is an important barrier against influenza. Understanding the mechanism of the human body’s response to influenza viruses can help transform influenza viruses into ideal oncolytic viruses. When the influenza virus infects the human body, it is first cleared by the mucus of the respiratory epithelium. After the influenza virus has passed through the mucus layer infects immune cells or non-immune cells, its viral RNA is recognized as a foreign substance and stimulates the body's secretion of IFN-I, proinflammatory cytokines, etc. Additionally, IFN produced by macrophages and DCs will promote the expression of hundreds of antiviral genes in the surrounding cells of the infected cell [92]. The virus protein will then be degraded into peptide fragments in antigen-presenting cells (APCs). These peptide fragments will be presented to T lymphocytes by MHC class I or class II molecules to stimulate the proliferation and differentiation of T lymphocytes into effector T cells and memory T cells. Effector T cells are responsible for eliminating infected cells and secreting antiviral cytokines. Memory T cells are responsible for reimmunization responses [93]. Infected cells will also become the target of NK cells. Finally, innate immunity, humoral immunity and cellular immunity are used to jointly construct the body's antiviral state.

### Antitumor mechanism of influenza virus

**Fig. 6:**
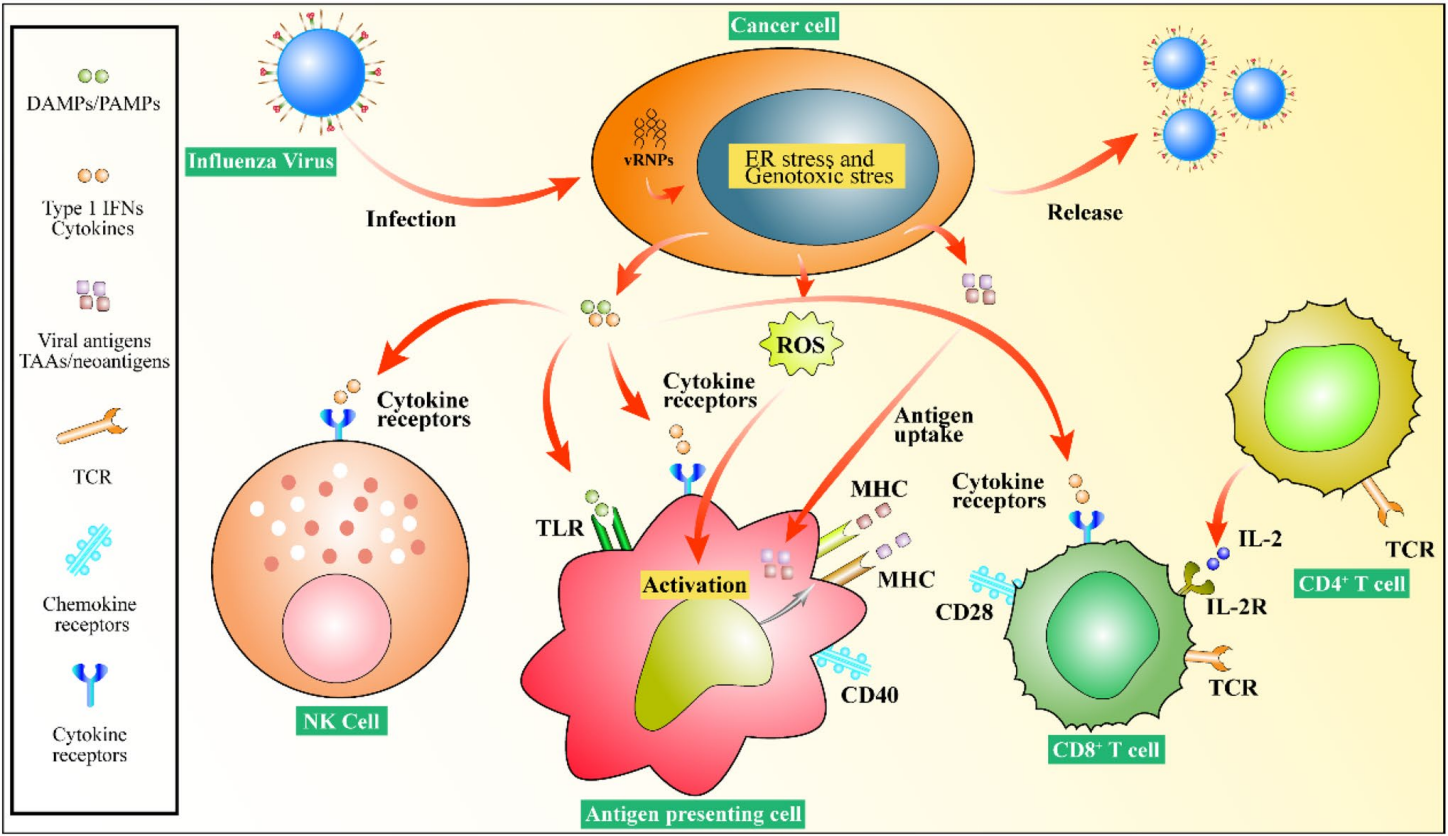
Influenza viruses infect tumor cells and elicit an antitumor immune response. Influenza viruses can induce the release of DAMPs/PAMPs, type 1 IFNs, viral antigens, and TAAs/neoantigens after infecting tumor cells. These cytokines will activate NK cells, APCs. and T lymphocytes to strengthen the immune response against the tumor and achieve tumor treatment

After the influenza virus infects tumor cells, several methods of killing the tumor cells are observed. First, the virus can proliferate in the cell and lyse the tumor cells. The virus particles released after cell lysis can infect other cells again until all the tumor cells are killed [85, 94, 95]. Second, influenza viruses express proteins that are cytotoxic to tumor cells during the cell replication cycle, and they also stimulate the body to produce tumor cell-specific and non-specific immune responses [96]. Tumors can escape the body's immunity largely because tumor cells can reduce the expression of major histocompatibility antigens and their stimulating factors so that the body cannot produce an effective immune response. An influenza virus constructed by reverse genetic technology can express some proteins on the surface of tumor cells after infecting the tumor cells, thereby stimulating the body's immune system to recognize the tumor cells and generate an immune response against them [85, 97] (Fig. 6).

### Insufficient antitumor applications of influenza virus

Modified influenza viruses still present certain problems when applied for tumor treatment. First, influenza viruses can only infect a portion of tumor cells. Therefore, designing a virus treatment plan that promotes the killing of adjacent uninfected cells will be a key point of influenza virus antitumor treatment. In addition, the safety of influenza viruses is also an important consideration. Although influenza virus is the most common human virus, from a safety perspective, the potential side effects caused by injecting live virus and returning non-disease-induced virus strains to a more pathogenic phenotype are controversial. In addition, due to the harsh tumor microenvironment, directly achieving tumor targeting is not always possible. These issues represent future challenges for influenza virus antitumor treatment.

### Future directions of influenza virus antitumor applications

Antitumor research on influenza virus has made great progress in recent years with the development of reverse genetic technology. First, in terms of tumor immunotherapy, multiple recombinant influenza viruses expressing tumor necrosis factor and interleukin family members have been established, and they have good therapeutic effects on tumor models [95, 98–100].

In addition, treating tumors by targeting their own immune characteristics represents a new direction for recombinant influenza viruses. Tumor antigens need to be degraded into short peptides within APCs and then the antigen peptide MHC-TCR complex is formed and recognized by T cells to stimulate CTL responses. Therefore, when designing a cancer treatment plan, whether it will induce a cytotoxic immune response against cancer cells should be determined. Tumor-associated antigens (TAAs) are effective targets for immune response induction. The construction of an influenza virus vector expressing a TAA through reverse genetic technology can effectively induce a specific immune response to the TAA, thereby inducing the body's own immune system to target the tumor to achieve treatment [96, 101, 102]. DCs are currently the most powerful APCs in the body. In recent years, increasing evidence has shown that the cellular immunity activated by DCs, especially the T cell-mediated CTL response, plays a leading role in the body's antitumor activity [103–105]. Influenza virus vectors load foreign gene fragments to infect DCs and induce cellular immune responses. Using DCs to induce CTLs is an effective method for tumor immunotherapy. DCs that have been genetically modified to express TAAs can effectively induce antitumor immune responses and have potential cancer treatment capabilities [96].

In recent years, additional tumor immunotherapy methods have been applied, and activated macrophages represent an important treatment option. Additionally, with the deepening of research on influenza virus targeting of tumors, oncolytic activity may be increased. At present, with the continuous development of cancer drug research and development, the combined use of recombinant viruses and antitumor drugs has also become a new antitumor method [106].

## Conclusions

In summary, reverse genetic manipulation technology has been used to redirect influenza viruses into viral vectors, which has good application prospects in clinical treatment and vaccine development. The oncolytic properties of influenza virus as well as its gene delivery vector ability can be applied to tumor therapy, including tumor vaccines and immunotherapy. Although the influenza virus poses a great threat to humans and has some problems in its application, continuous improvements in its transformation may promote the beneficial effects of this virus.
